# Rhabdovirus Matrix Protein Structures Reveal a Novel Mode of Self-Association

**DOI:** 10.1371/journal.ppat.1000251

**Published:** 2008-12-26

**Authors:** Stephen C. Graham, René Assenberg, Olivier Delmas, Anil Verma, Alireza Gholami, Chiraz Talbi, Raymond J. Owens, David I. Stuart, Jonathan M. Grimes, Hervé Bourhy

**Affiliations:** 1 Division of Structural Biology and Oxford Protein Production Facility, Wellcome Trust Centre for Human Genetics, University of Oxford, Oxford, United Kingdom; 2 UPRE Lyssavirus Dynamics and Host Adaptation, WHO Collaborating Centre for Reference and Research on Rabies, Institut Pasteur, Paris, France; Wake Forest University School of Medicine, United States of America

## Abstract

The matrix (M) proteins of rhabdoviruses are multifunctional proteins essential for virus maturation and budding that also regulate the expression of viral and host proteins. We have solved the structures of M from the vesicular stomatitis virus serotype New Jersey (genus: *Vesiculovirus*) and from Lagos bat virus (genus: *Lyssavirus*), revealing that both share a common fold despite sharing no identifiable sequence homology. Strikingly, in both structures a stretch of residues from the otherwise-disordered N terminus of a crystallographically adjacent molecule is observed binding to a hydrophobic cavity on the surface of the protein, thereby forming non-covalent linear polymers of M in the crystals. While the overall topology of the interaction is conserved between the two structures, the molecular details of the interactions are completely different. The observed interactions provide a compelling model for the flexible self-assembly of the matrix protein during virion morphogenesis and may also modulate interactions with host proteins.

## Author Summary

Rhabdoviruses are of considerable socioeconomic importance. For example, rabies virus causes lethal encephalitis resulting in approximately 50,000 human deaths per year. Rhabdoviruses infect cells and propagate despite having small genomes that encode only five multifunctional proteins. One of these, the matrix protein, plays a structural role in virus assembly in addition to modulating the production of host and virus proteins, promoting viral egress from the host cell and modulating cell death. We have solved the 3-dimensional crystal structures of matrix proteins from two distantly related rhabdoviruses: Lagos bat virus and vesicular stomatitis virus. The two proteins have very similar structures despite having dissimilar amino acid sequences. Surprisingly, for both we observe self-association between a pocket on the main globular domain and one extremity of an adjacent molecule in the crystal. Repetition of this interaction gives rise to non-covalent polymers of matrix proteins, adjacent proteins being tethered by a flexible linker. This provides a compelling molecular mechanism for the self-association of matrix molecules required for virus assembly. While the general mode of polymerization is conserved between the two structures, the precise molecular details of the interactions differ, consistent with these matrix proteins binding different cellular factors during infection.

## Introduction

Rhabdoviruses are single-stranded RNA viruses that possess non-segmented negative-sense genomes encoding five open reading frames and form enveloped, bullet-shaped virions [1]. Dimarhabdoviruses, the supergroup of rhabdoviruses that infect mammals and mosquitoes [2], are of considerable economic and social importance. Members of the genus *Lyssavirus* such as rabies virus cause lethal meningoencephalitis in humans and animals [3] while vesicular stomatitis virus (VSV; genus *Vesiculovirus*) causes symptoms clinically identical to those of foot-and-mouth disease in cattle and occasional, limited infections in humans [1].

The rhabdovirus matrix (M) protein is small (∼20–25 kDa) but plays a number of roles during the replication cycle of the virus. The M protein is an important structural component of rhabdovirus virions, forming a layer between the glycoprotein- (G-) containing outer membrane and the nucleocapsid core composed of the virus nucleoprotein (N), polymerase (L), phosphoprotein (P) and RNA genome [4]–[6]. M condenses the nucleocapsid core into the ‘skeletons’ seen in mature virions [7],[8], recent evidence suggesting that M does so in association with pre-formed nucleocapsid–G plasma membrane microdomains [9]. M aggregates *in vitro* [10]–[12] to form long fibers [11], the N terminus of the protein and a region between residues 121–124 being important for this self-association [11],[13]. In addition to being spread through the cytosol of infected cells, M is targeted to mitochondria [14],[15], to nuclei [16], and to plasma membranes [9],[17]. M has been shown to interact directly with negatively-charged membranes [17]–[19] and can induce their deformation [20]. This interaction is mediated primarily by the N terminus of M, which contains several positively-charged amino acid residues, although in VSV residues 121–124 may also be involved [19]–[22].

In addition to its structural roles, M has been implicated in controlling the balance between transcription and replication of the viral genome [21],[23], in promoting budding [24],[25], and in modulating host-cell transcription [26], -translation [27] and -apoptosis [28]–[31]. The specific protein∶protein interactions that mediate a number of these functions have been identified. A ‘late domain’ (sequence PPXY) located toward the N terminus of M promotes budding by interacting with the WW-domain of NEDD4, a ubiquitin ligase that interacts with the vesicle formation and cargo sorting ESCRT complexes, although the precise mechanism by which this would facilitate budding remains unclear [24],[25],[32],[33]. VSV M has been shown to inhibit the production of host proteins by binding directly to Rae1 and blocking the export of host mRNA from the nucleus, residues near the N terminus of M being essential for this interaction [27],[34]. M also regulates the translation of host mRNA by binding to and/or modulating the phosphorylation state of translation initiation factors [26],[35],[36], as well as inducing the production of viral proteins through an unknown mechanism [21].

To date, the only structural information available on rhabdovirus M proteins is the structure of a thermolysin-stable M core (M^th^) of VSV Indiana (VSV_Ind_) [19]. The proteolytic treatment removed the N-terminal 47 residues and cleaved the surface-exposed hydrophobic loop between residues 121–124, and only residues 58–121 and 128–227 were visible in the structure. To further investigate the role of the N terminus and hydrophobic surface-exposed loop and to investigate the structural conservation of M across *Rhabdoviridae*, we solved the structures of full-length M from VSV serotype New Jersey (VSV_NJ_) and from the lyssavirus Lagos bat virus (LBV). These structures reveal that rhabdovirus M proteins share a similar overall fold and self-associate via a stretch of amino acids (in the otherwise-disordered N terminus) that bind to a similar region on the globular domain, although the molecular details of the interaction interface differ dramatically between VSV_NJ_ and LBV M. This inter-molecular interaction provides a plausible mechanism for the self-assembly of M, leading to enhanced affinity for membranes. Further, the differences in this interaction provide a structural framework for understanding the distinct cytopathic effects of vesiculoviruses and lyssaviruses.

## Results

### Structure of M from VSV serotype New Jersey (VSV_NJ_)

**Figure 1:**
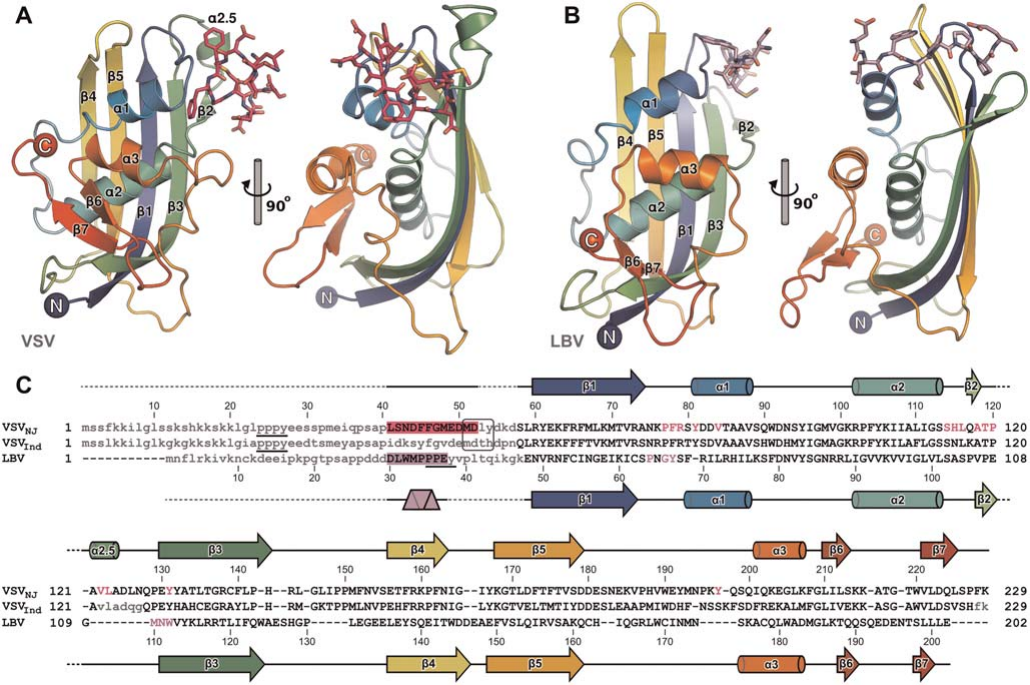
The structure of the matrix (M) proteins from vesicular stomatitis virus New Jersey (VSV_NJ_) and Lagos bat virus (LBV). The globular domains of (A) VSV_NJ_ M (residues 58–229) and (B) LBV M (residues 48–202) are shown in two orthogonal views, color ramped from blue (N terminus) to red (C terminus). The residues from the N-terminal regions that interact with the globular domains show as sticks (VSV residues 41–52, pink; LBV residues 30–37, violet). (C) Structure-based alignment of the M sequences from VSV_NJ_, VSV_Ind_ and LBV. The residues from the N termini that interact with the globular domains are highlighted and residues with which they interact are in colored typeface. The PPXY ‘late domains’ of LBV and VSV M are underlined and residues of VSV M proposed to interact with Rae1 are boxed. Secondary structure is shown above/below the relevant sequences with β-sheets, α-helices and polyproline-II helices are shown as arrows, cylinders and triangular prisms, respectively. Residues not observed in the structures are not aligned and are shown as grey lower-case text.

The structure of VSV_NJ_ M was solved by SeMet single-wavelength anomalous dispersion phasing and refined to 1.83 Å resolution with residuals R/R_free_ = 0.157/0.179 (Table 1). VSV_NJ_ M forms a globular domain with a central α helix sandwiched on one side by an extensive 5-stranded β sheet and on the other by two α helices and a smaller two-stranded β sheet (Figure 1A) that is very similar to the thermolysin-resistant core of M (M^th^) from VSV serotype Indiana (VSV_Ind_), with 0.9 Å root-mean-squared displacement (rmsd) over 156 C^α^ atoms. Residues 121–128, disordered in the VSV_Ind_ M^th^ structure, are observed in the structure of VSV_NJ_ M with residues 121–124 forming a short stretch of α helix (α2.5, Figure 1A). The N-terminal 57 residues of VSV_NJ_ M do not form part of this globular domain. While residues 1–40 and 53–57 are not well ordered and could not be modeled in electron density, residues 41–52 are located in strong electron density bound in a deep hydrophobic pocket formed by the loops between sheet β1 and helix α1, the region between helices α2 and α2.5 (including sheet β2), and the stretch of 3–10 helix immediately preceding helix α3.

**Table 1:** Data Collection and Refinement statistics.

Data collection statistics	*VSV*	*Lagos Bat*
Wavelength (Å)	0.980	0.873
Resolution limits (Å)^a^	35–1.83 (1.88–1.83)	40–2.75 (2.82–2.75)
Space group	*I*4	*P*6_1_22
Unit cell dimensions (Å, °)	*a* = *b* = 86.3, *c* = 70.3	*a* = *b* = 56.9, *c* = 187.9
Unique reflections^a^	22,813 (1,697)	5,164 (367)
Redundancy a	7.3 (6.1)	6.2 (5.4)
Completeness (%) a	99.8 (99.6)	99.6 (99.4)
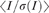 a	10.7 (2.0)	10.2 (2.2)
*R*_merge_ (%) a ^, ^ b	0.125 (0.844)	0.128 (0.769)
**Refinement statistics**		
Resolution limits (Å) a	34–1.83 (1.88–1.83)	35–2.75 (2.82–2.75)
Number of reflections in working set a	21,673 (1,603)	4862 (343)
Number of reflections in test set a	1,130 (85)	291 (19)
Wilson *B* (Å^2^)	21.1	59.7
*R* factor of working set (%) a ^, ^ c	0.157 (0.266)	0.207 (0.277)
*R*_free_ (%) a ^, ^ c ^, ^ d	0.179 (0.244)	0.255 (0.376)
Number of atoms (protein/water)	1,483 / 147	1,320 / 0
Number of atoms with alternate conformations (protein/water)	23 / 0	0 / 0
Residues in Ramachandran favoured region (%)	98.3	96.9
Ramachandran outliers (%)	0.0	0.0
r.m.s.d bond lengths (Å)	0.011	0.009
r.m.s.d bond angles (°)	1.249	1.093
Average *B* factors (Å^2^) (globular domain / ‘ligand’ / water)	23.5 / 51.1 / 24.6	46.8 / 85.7 / −

a Numbers in parentheses are for the highest resolution shell.

b *R*_merge_ = Σ_hkl_Σ_i_|*I(hkl;i)*−〈*I(hkl)*〉|/Σ_hkl_Σ_i_ *I(hkl;i)*, where *I(hkl;i)* is the intensity of an individual measurement of a reflection and 〈*I(hkl)*〉 is the average intensity of that reflection.

c *R* = Σ_hkl_||*F*_obs_ *(hkl)*|−|*F*_calc_ *(hkl)*||/Σ_hkl_|*F*_obs_ *(hkl)*|, where |*F*_obs_ *(hkl)*| and |*F*_calc_ *(hkl)*| are the observed and calculated structure facture amplitudes.

d *R*_free_ equals the *R*-factor of test set (5% of the data removed prior to refinement).

**Figure 2:**
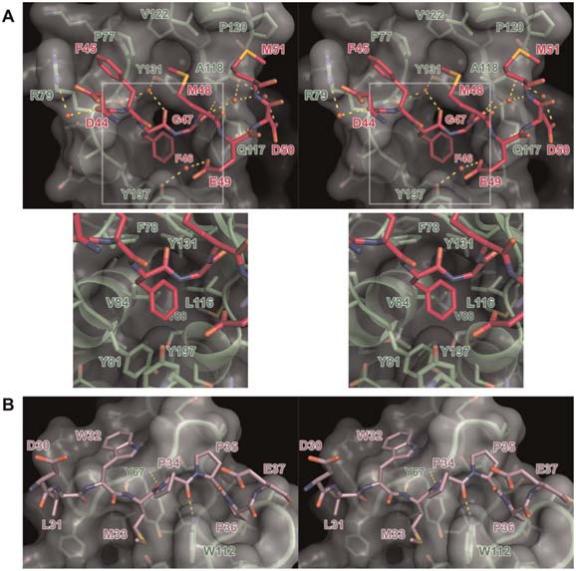
Molecular details of the self-association interfaces differ significantly between VSV_NJ_ and LBV M. Stereograms of the interactions between the N-terminal regions of (A) VSV_NJ_ (residues 44–51, carbon atoms pink) and (B) LBV (residues 30–37, carbon atoms violet) with the globular domains of the respective proteins (carbon atoms green). Hydrogen bonds are shown as yellow dashed lines and molecular surfaces of the globular domains are shown in white. For clarity, in (A) only solvent atoms and residues of the N-terminal segment that directly mediate the interaction are shown, and an inset (below) shows the residues that line the deep hydrophobic pocket bound by F46.

F46 is central to the interaction: the side chain of F46 sits deep in the hydrophobic pocket lined by the side chains of residues Y81, V84, L116, Y131, Y197 and the backbone between residues 114 and 116 (Figure 2). The backbone of F46 forms hydrogen bonds with the backbone of F78 and with a water molecule that bridges the backbone of F78 and side chain of Y131. Residues 45–51 of the bound ligand interact with residues flanking the deep pocket into which the side chain of F46 binds. F45 sits in a shallow hydrophobic pocket lined by the hydrophobic side of the backbone peptide planes between residues 77–79 and by the side chains of P77 and R79. The backbone of G47 forms H-bonds with the backbone of A118 and with a solvent atom that bridges the backbones of residues G47, A118 and M51. M48 sits in a shallow groove formed by the side chains of P77, A118, V122 and Y131. E49 forms a hydrogen bond with the side chain of Q117 and with a water molecule that also hydrogen bonds with the side chain of Y197. The backbone of D50 forms a hydrogen bond with the side chain of Q117. M51 sits in a shallow hydrophobic pocket formed by the hydrophobic side chains of A118, P120, V122, L123 and the hydrophobic peptide plane between residues 118 and 120. Overall, these interactions bury 1050 Å^2^ of surface area. The co-localization of strong anomalous scattering with the two SeMet residues (48 and 51) allowed unambiguous identification of the bound peptide (Figure S1A) and SDS-PAGE analysis confirmed that the crystallized VSV_NJ_ M was intact (Figure S2A).

**Figure 3:**
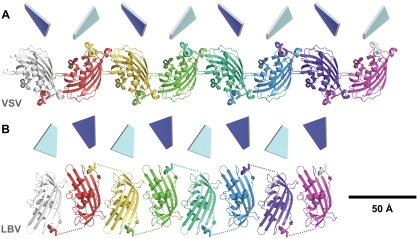
VSV_NJ_ and LBV M form non-covalent linear polymers *in crystallo*. The interactions between N-terminal segments and globular domains of (A) VSV_NJ_ M and (B) LBV M that give rise to the non-covalent linear polymers observed in the crystals are shown. Quadrilaterals show the relative orientations of the extensive 5-stranded β sheets, the faces representing the helix-facing (front, dark blue) and solvent-exposed (back, light blue) sides of the sheet.

Residue 52 of the bound peptide is 46 Å from the first ordered residue of the globular domain to which it binds (S58), a distance too great to be spanned by the missing 5 residues. Distance constraints dictate that this bound peptide derives from an adjacent molecule in the crystal, related by the crystallographic symmetry operator [−*x*−½, −*y*−½, *z*−½] with residue 58 lying 11 Å from residue 52 of the bound peptide (C^α^–C^α^ distance). While electron density linking residue 52 to residue 58 of the adjacent monomer is observed in maps calculated using data to 4 Å resolution, these residues could not be modeled because in higher resolution maps this density is significantly reduced, presumably due to disorder. The interaction of the globular domain with the N-terminal peptide of an adjacent molecule in the crystal gives rise to non-covalently linked linear polymers of VSV_NJ_ M monomers (Figure 3A).

**Figure 4:**
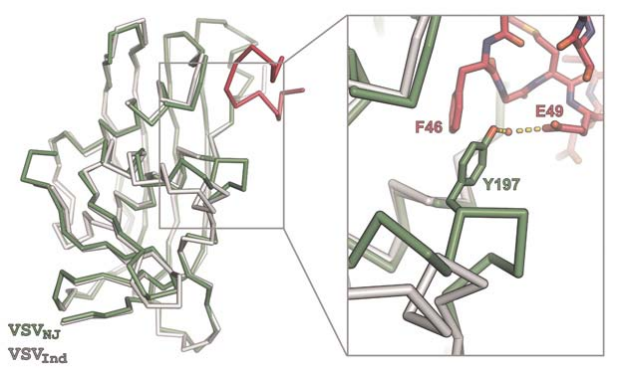
The orientations of residues 191–202 differ significantly between VSV_NJ_ M and VSV_Ind_ M^th^. Backbone C^α^ traces of VSV_NJ_ M (green and pink) and VSV_Ind_ M^th^ (grey) are shown. The inset shows the side chain of Y197, which forms part of the hydrophobic pocket in which F46 sits and also forms a water-mediated hydrogen bond with E49 from the bound N-terminal segment.

Asides from the bound N terminus, the most striking structural difference between VSV_NJ_ M and VSV_Ind_ M^th^ in the globular domain is in the orientation of residues 191–202, which form part of helix α3 and of the loop that precedes it (Figure 4). In VSV_NJ_ M this loop has shifted toward the interacting N-terminal residues: the C^α^ atom of K196 moves 9.5 Å from its position in VSV_Ind_ M^th^ and residues 195–200 form a stretch of 3–10 helix. Y197 on this helix forms part of the deep hydrophobic pocket in which F46 resides, in addition to forming a water-mediated H-bonds with the side chain of E49 (Figure 4).

**Figure 5:**
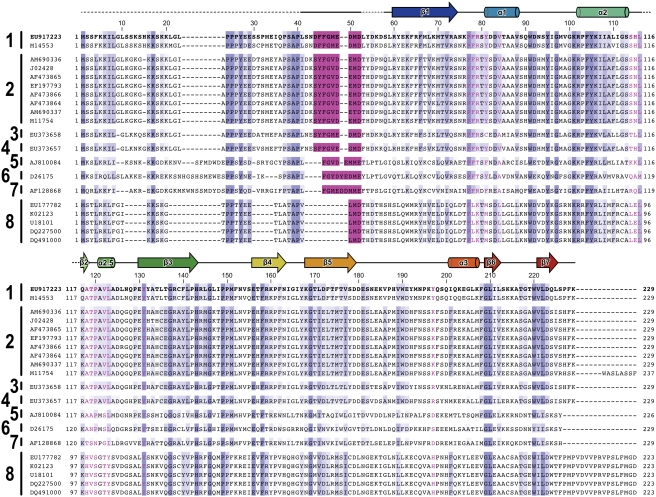
Sequences of vesiculovirus and spring viremia of carp virus M. Sequences of M from the following vesiculovirus/dimarhabdovirus serotypes/genera are shown: 1, VSV New Jersey; 2, VSV Indiana; 3, Alagoa virus; 4, Cocal virus; 5, Isfahan virus; 6, Chandipura virus; 7, Piry virus; and 8, Spring viremia of carp virus. GenBank accession IDs for all sequences are shown and the sequence used for structure determination is in bold typeface. Residues that are highly or moderately conserved (BLOSUM62 scoring) are colored marine and light blue, respectively. Residues from the N-terminus that interact with the globular domain are colored pink and residues that they interact with are in pink typeface. Secondary structure is shown above the sequences (β-sheets and α-helices are shown as arrows and cylinders, respectively).

Alignment of vesiculovirus M sequences (Figure 5) shows that F46 and the residues that form the deep binding pocket in which it sits are highly conserved amongst VSV serotypes, although there are some conservative substitutions of flanking residues. In viruses Isfahan, Piry, Alagoa, Cocal and Chandipura the core F46 residue of the N-terminal interacting motif is conserved, but the flanking residues are significantly changed. However, side chains that form the hydrophobic pocket in which F46 is buried are conserved (Y131, F78) or conservatively substituted (Y81F/C, V84A, L116M). In spring viremia of carp virus, a dimarhabdovirus [2] not assigned to the vesiculovirus genus that infects fish rather than mammals, residue F46 is not conserved and it is unclear whether M from this virus would be able to self-associate in a manner similar to that observed for VSV_NJ_ M.

### Structure of M from Lagos bat virus (LBV)

The structure of LBV M was solved by SeMet multi-wavelength anomalous dispersion phasing and refined to 2.75 Å resolution with residuals R/R_free_ = 0.207/0.255 (Table 1). Residues 48–202 of LBV M form a globular domain with an overall fold that closely resembles those of VSV_Ind_ M^th^ and VSV_NJ_ M, with 2.8 and 3.1 Å root-mean-squared deviation between 138 and 139 equivalent C^α^ positions, respectively, despite the aligned residues sharing less than 10% sequence identity (Figure 1). While the central α helix and back 5-stranded β sheet overlay very well, the loop between sheets β2 and β3 is much shorter in LBV M than in VSV_NJ_ M and no stretch of helix is present between these sheets. Helices α1 and α3, sheets β6 and β7 and the loop between sheets β4 and β5 are also shifted between the LBV M and VSV_NJ_ M structures.

**Figure 6:**
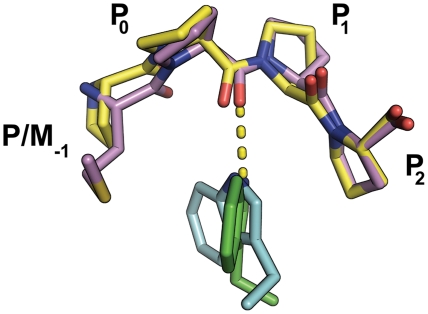
The LBV M self-association represents a novel proline-rich motif (PRM) binding its cognate PRM–binding groove. Residues 33–36 of LBV M (sequence MPPP, violet carbon atoms) wrap around a central tryptophan residue (W112, green carbon atoms) on the surface of the globular domain of LBV M in a manner similar to that of a PPPP peptide (yellow carbon atoms) bound to the PRM-binding domain of EVH1 (cyan carbons; PDB ID 1EVH [73]). In both cases, the carbonyl oxygen of P_0_ forms a hydrogen bond with the central tryptophan residue, this tryptophan also forming hydrophobic interactions with P/M_−1_ and P_2_.

Strikingly, in the structure of LBV M a stretch of peptide is again observed bound in a shallow hydrophobic groove formed by the β1–α1 and β2–β3 loops of the globular domain (Figure 1B). Anomalous difference density co-located with the Se atom of SeMet33 in the SeMet-labelled protein unambiguously identifies the bound peptide as LBV M residues 30–37 (Figure S1B), no electron density being evident for residues 1–29 or 38–47. This interaction is centred on residues 32–36, which form a short stretch of left-handed polyproline-II helix (Figure 2B). The backbone of residues 33–35 packs against the backbone of the β1–α1 loop of the globular domain, M33 forming two hydrogen bonds with Y67. W112 in the β2–β3 loop sits under residues 33–35, its indole nitrogen forming a hydrogen bond with the carbonyl oxygen of P34 (Figure 6). The side chain of P36 sits in a hydrophobic pocket formed by the P107 and W112 side chains and the backbone of M110 and N111 (Figure 2B). Overall, these interactions bury 830 Å^2^ of surface area. SDS-PAGE confirmed that the crystals contained full-length LBV M (Figure S2B) and gel filtration analyses of full-length and truncated (Δ1–45) LBV M were consistent with the N-terminal portion of M adopting an extended/disordered conformation in solution (Figure S3) as has been observed previously for VSV M [13]. As in VSV_NJ_, the distance between the last residue of the bound peptide and the first residue of the LBV M globular domain is too great to be spanned by the intervening residues (49 Å from E37 C^α^ to E48 C^α^), and the bound peptide must come from a neighbouring monomer. The interaction with the most likely monomer, related by the crystallographic symmetry operator [1+*x*−*y*, 1−*y*, 1−*z*] (23 Å from E37 C^α^ to E48 C^α^; Figure S4), generates linear polymers of non-covalently linked molecules in the crystal (Figure 3B).

**Figure 7:**
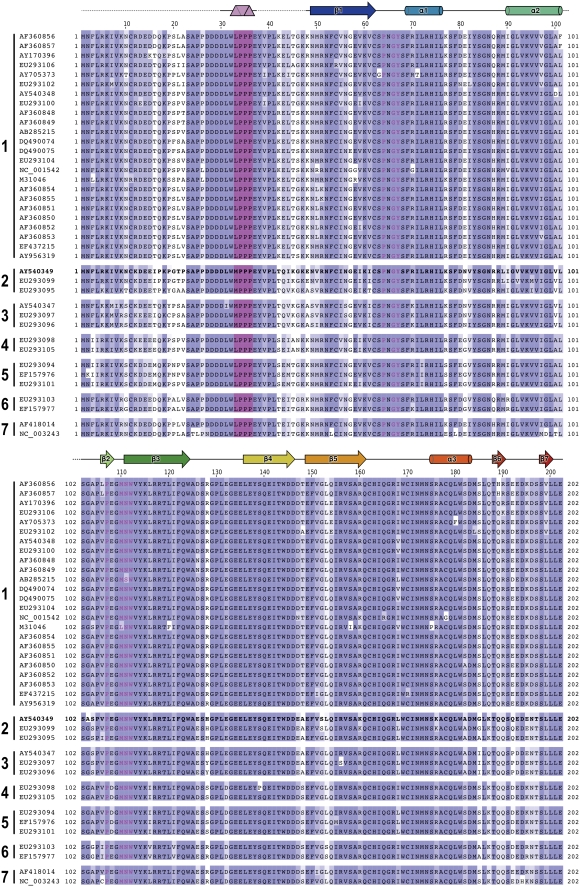
Sequences of lyssavirus M. Sequences of M from the following lyssavirus genotypes are shown: 1, Rabies virus; 2, Lagos bat virus; 3, Mokola virus; 4, Duvenhage virus; 5, European bat lyssavirus 1; 6, European bat lyssavirus 2; 7, Australian bat lyssavirus. Sequences presented are representative of the diversity of lyssavirus M proteins. GenBank accession IDs for all sequences are shown and the sequence used for structure determination is in bold typeface. Residues that are highly or moderately conserved (BLOSUM62 scoring) are colored marine and light blue, respectively. The [M/L]PPP proline-rich motif is colored purple and residues that interact with this motif are in purple typeface. Secondary structure is shown above the sequences (β-sheets, α-helices and polyproline-II helices are shown as arrows, cylinders and triangular prisms, respectively).

The sequence of the interacting N-terminal region of LBV M is conserved in lyssaviruses, the only exception being the conservative substitution of M33 with leucine (Figure 7). The residues of the globular domain to which they bind are also conserved, with just two non-disruptive exceptions (M110L in strain SADB-19 and N111S in strain ZAMRAV51, Figure 7).

## Discussion

### The M proteins of VSV_NJ_ and LBV self-associate in a similar but distinct manner

In the structures of both VSV_NJ_ and LBV M, a peptide from the otherwise-disordered N terminus of the protein is observed bound to the globular domain near the β1–α1 and β2–β3 loops (Figures 1A & 1B). The overall location of this interacting region in the sequence of the proteins is similar, the interacting residues being less than 20 residues from the start of the globular domain (Figure 1C), and in both the interaction is between adjacent molecules, thereby forming non-covalently linked linear polymers of M *in crystallo* (Figure 3). It is therefore particularly striking that the nature of the interfaces formed by the N-terminal interacting residues and the globular domains differ so significantly (Figure 2). The similar overall nature of the self-association, despite large differences in the molecular details of the interaction interfaces, is compelling evidence that the self-interaction is biologically relevant rather than being an artefact of crystallization.

The self-association of VSV_NJ_ M is centered on F46, which binds into a deep hydrophobic pocket on the surface of the globular domain. Residues 45–47 adopt an extended conformation, flanked on either side by single turns of α helix. To the best of our knowledge this self-association interface shares no homology with previously identified protein∶protein interaction interfaces. In contrast, the peptide recognition cleft of LBV M is quite shallow and residues 33–36 (sequence MPPP), which bind into this hydrophobic pocket on LBV M, form a short stretch of polyproline-II helix. The recognition of polyproline-II helices formed by proline-rich motifs (PRMs) is an important theme in protein∶protein interactions. Six classes of PRM-binding domains have previously been described: SH3, WW, EVH1, UEV and GYF domains and profilins [37]. These are generally characterized by the presence of a central tryptophan residue, around which the polyproline helix wraps, with shallow hydrophobic pockets accommodating the proline side chains [37]. LBV M exhibits this generic mode of binding (Figure 6), but details of the interaction differ from the known classes of PRM interactions. The structure of LBV M therefore reveals both a novel 7^th^ family of PRM-binding domain and defines the interaction of this domain with its cognate ligand.

### The role of M self-association in rhabdovirus assembly

The M proteins of rhabdoviruses play important roles in virus assembly. They condense the nucleocapsid cores into a tightly-coiled nucleocapsid-M complex (termed ‘skeletons’) [7],[8], form a layer between the nucleocapsid and the surrounding lipid bilayer [4]–[6], and promote virus budding [6], [24], [38]–[40]. An obvious functional implication of the self-association in the structures of VSV_NJ_ and LBV M is in virus assembly, by facilitating long-range organization of M molecules and thereby enhancing the local concentration of M.

Experiments investigating the self-association of VSV M support this hypothesis. M assembly is a two-stage process involving the sequential addition of M monomers to small pre-formed M nuclei to form fibres [10],[11]. The N-terminal portion of M plays a critical role in the second step, polymerisation [11],[13]. Treatment of M with trypsin gives rise to a stable fragment (M^t^) spanning residues 44–229 [41] that retains the ability to form fibres but can only nucleate M aggregation in the presence of added Zn^2+^ [10],[11]. However, M treated with therymolysin (M^th^), comprising residues 48–121 and (122, 123 or 124)–229, does not aggregate [13],[19]. This is consistent with removal in M^th^ of F46, the residue central to the interaction, and with the ‘untethering’ of the β2–β3 loop, which forms part of the interaction surface on the globular domain (Figures 2 & S5). We propose that the association between the N-terminal portion of M and the globular domain of an adjacent M molecule observed in our structures is the same as that which promotes addition of M monomers to pre-formed nuclei to yield large M fibers *in vitro* [11] and presumably promotes virus assembly *in vivo*. While it is possible that the movement of the loop that links sheet β5 to helix α3 in VSV_NJ_ M relative to VSV_Ind_ M (Figure 4) represents a conformational switch that facilitates nucleation or polymerization, the mechanism by which such a switch would be induced remains unclear.

Mutational analysis of the β2–β3 loop supports a role for the observed interaction between the N-terminal portion of M and the globular domain of an adjacent molecule in virus morphogenesis. Substitution of VSV_Ind_ residues 121–124 (sequence AVLA) with DKQQ gives rise to a mutant M protein that shows reduced capacity to recruit free M or M_DKQQ_ into pre-formed nucleocapsid-M_DKQQ_ complexes, although the general ability to self-associate is maintained [21]. Mapping this mutation onto the structure of VSV_NJ_ M (Figure S5) reveals that the mutated residues surround the site of interaction of the N-terminal peptide, but they do not interfere with the burial of F46 into the deep hydrophobic pocket and would thus presumably not abolish the interaction entirely. A double mutation of M48 and M51 to arginine in VSV_NJ_ yields a virus phenotype competent for assembly but unable to inhibit host-cell gene expression [42]–[44]. The side chain of M48 is not required for self-association, since in VSV_Ind_ it is replaced with valine (Figure 5). Further, while both M48 and M51 form part of the VSV_NJ_ self-association interface, neither is completely buried (Figures 2 & S5) and the observed interaction could most likely be maintained in the mutated M protein with little energetic penalty.

Mutation of residues 35 and 36 in the N-terminal interacting region of lyssavirus M to serine and alanine, respectively, reduces viral fitness [45]. This is consistent with our proposed model, although further experiments are required to distinguish mutations that modulate self-association from those which interrupt the interaction of the PPXY ‘late domain’ with the host-cell budding machinery.

Assembly of rhabdoviruses may require the M protein to interact with a circular or helical scaffold formed by the nucleocapsid [46]–[49]. As the non-covalent polymers of M observed in the crystal lattices are straight, the relative orientations of adjacent globular domains observed in the crystals might not reflect the packing of globular domains in the final assembled virion. However, the flexible nature of the tether that links one globular domain to the next, via interaction with the flexible N-terminal segment, could accommodate major rearrangements of adjacent globular domains. This would allow the required curvature of the M polymers and facilitate reorientation of M to interact with other components of the virion. Based on the dimensions of ‘shaved’ VSV virus particles and number of M molecules in such particles [50], assuming a model where M lies immediately below the plasma membrane, the mean distance between the centres of adjacent M proteins would be ∼45 Å. This is significantly larger than the M-to-M distances observed in the non-covalent linear polymers formed within the crystals (35 Å for VSV_NJ_ and 28 Å for LBV, Figure 3) and is consistent with a loose tethering of M proteins in the assembled virions. Such flexibility would allow for higher concentrations of M at points of higher membrane curvature, as has been observed recently for VSV M [9]. A similar beads-on-a-string arrangement in virions has been postulated for the influenza virus matrix protein [51].

### Self-association enhances the affinity of VSV M for membranes

The self-association of M identified in our structure informs previous experiments on the association of VSV M with membranes. M associates with membranes both *in vitro* [18]–[20] and *in vivo* [9],[22]. M is thought to link the nucleocapsid and the envelope of the virus [4]–[6], although recent evidence suggests that M might actually be recruited to pre-formed nucleocapsid–G plasma membrane microdomains [9]. M^t^, in which the N-terminal 43 residues are removed by trypsin proteolytic cleavage, maintains its ability to interact with membranes [19],[20]. The interaction is significantly weaker than for wild-type M, confirming that the positive-charge of the lysine-rich N terminus is important for membrane association, but the fact that some association is maintained suggests the presence of other, potentially weaker membrane-interaction interfaces on M [22]. In contrast, M^th^ is almost entirely unable to interact with membranes [19],[20]. M^th^ has only 4 residues fewer at the N terminus than M^t^ [13]. Since none of these are positively-charged, the decrease in affinity can't be due to a loss of charge-mediated affinity for membranes. Furthermore, substitution of the hydrophobic side chains in the surface loop cleaved by thermolysin (residues 121–124) for charged residues does not abolish membrane association [21], indicating that an additional membrane-attachment interface has not been excised by the thermolysin treatment. We propose that the observed decrease in membrane affinity arises instead from the inability of M^th^ to self-associate. Polymerisation at the membrane, mediated by the self-association observed in our structure, would provide avidity enhancement of binding thus overcoming the lack of N-terminal charge in M^t^.

### Interaction of M with cellular proteins

In addition to their role in virus assembly and budding, rhabdovirus M proteins are important for subverting the host immune response by suppressing the production of host genes. It has previously been observed that VSV M blocks host gene translation by binding directly and specifically to Rae1, a protein involved in nuclear export of mRNA [34]. Substitution in M of residues 52–54 with alanine completely abolishes this interaction [27],[34]. A second substitution in this area, M51R has the same effect, although a direct loss of interaction with Rae1 has not been shown for that mutant [52]. Our structure reveals that the Rae-1 binding site on VSV M partially overlaps with the N-terminal self-association motif (Figure 1). Steric considerations make it likely that self-association of VSV M and Rae-1 binding would be mutually exclusive.

A direct interaction between a cellular protein and a sequence overlapping the N-terminal portion of M involved in self-association has also been observed in lyssaviruses. In this case the interaction is between the ‘late domain’ (sequence PPEY, residues 35–38) and the WW domain of NEDD4 [24], a ubiquitin ligase that interacts with proteins in the ESCRT pathway and promotes virus budding (Figure 1) [25]. The polyproline-II helix conformation adopted by residues 32–36 of LBV M is entirely compatible with binding of this ‘late domain’ to the NEDD4 WW domain. As above, steric clashes would prevent simultaneous interaction of these residues with WW domains and with the globular domain of LBV M. Since PRMs and their binding motifs are such a common theme in cellular protein∶protein interactions it is likely that both the PRM motif and PRM-binding groove of LBV M also mediate specific interactions with other cellular proteins, although such binding partners are yet to be identified.

Such an interaction between the self-association pocket on the globular domain of M and an unknown cellular protein has recently been identified for VSV. While mutation of the VSV_Ind_ M β2–β3 loop residues 121–124 to DKQQ (M_DKQQ_) interferes only modestly with the self-association of M (see above), it produces a marked reduction in the amount of viral mRNA translated in infected cells [21]. As this phenotype can be rescued by co-infection with wild-type VSV it probably arises from a loss of function rather than a gain of inhibition. This phenotype was mapped specifically to the β2–β3 loop, reversion of residues 121–122 to the wild-type sequence (AV) restoring wild-type levels of viral mRNA translation [21]. It is likely that the yet-unidentified factor required to promote efficient viral mRNA translation binds in a manner similar to that observed for the N-terminal segment in our structure. As discussed above, V122 forms part of the binding pocket for M48 (V48 in VSV_Ind_). While mutation of V122 to lysine doesn't significantly impair self-association of VSV_Ind_ it is possible that the effect would be greater on ligands with a larger hydrophobic residue in a position equivalent to residue 48. It is equally likely that V122 would be more buried in an interaction with this (unknown) cellular partner, reducing its ability to ‘swing away’ from the binding cleft.

To summarise, both VSV and lyssavirus M have known cellular interaction motifs that overlap with the self-interaction motifs revealed by the structures presented here. However, the molecular details of the self-interaction motifs differ significantly and it is likely that the cellular binding partners of VSV and LBV M proteins are also distinct, consistent with the different host-range and cytopathogenicity of vesiculoviruses and lyssaviruses. The region between the β1–α1 and β2–β3 loops and the fragments of the otherwise-disordered N-terminal tails to which they bind are clearly hot-spots for rhabdovirus M protein∶protein interactions. This suggests a tempting evolutionary hypothesis to explain the similarity in overall interaction topology but difference in molecular interfaces between vesiculoviruses and lyssaviruses. The self-association grooves on the globular surfaces of the proteins and their cognate ligands might have evolved to mimic desirable protein∶protein interactions within the host cells, the functional constraint imposed by needing to remain competent for self-assembly (and thus enable viral morphogenesis) having maintained the overall topology of the interaction. The maintenance of interaction with cellular partners and self-association at the same locus on the protein raises a second interesting possibility, that the observed self-associations also play a role in regulating interaction of M with cellular partners by (partially) sequestering the binding interfaces, as has been suggested for the HIV M protein [53]. This would provide a *raison d'être* for the shortened M gene product observed in VSV (residues 51–229) [43]; it possesses the deep hydrophobic peptide-binding groove but not the N-terminal peptide, thereby providing the virus with a pool of M protein (with unoccupied binding grooves on their globular surfaces) able to interact with cellular binding partners.

## Materials and Methods

### Cloning, expression, purification, crystallization and data collection

The matrix (M) protein from Lagos bat virus (LBV) was cloned, expressed, purified and diffraction data were collected as described previously [54]. M from VSV serotype New Jersey (VSV_NJ_) was cloned into pOPINS, encoding an N-terminal His_6_-SUMO fusion tag, and selenomethionine-labelled (SeMet) protein was expressed and purified as described for LBV M [54]. Purified VSV_NJ_ M was concentrated to 1.2 mg/mL and crystallization trials were attempted at 20.5°C in sitting drops containing 100 nL protein and 100 nL precipitant solution equilibrated against 95 µL reservoirs in 96-well plates. Crystals of SeMet VSV_NJ_ M grew in 20% v/v isopropanol, 20% w/v PEG 4000 and 0.1 M sodium citrate (pH 5.6) and were cryoprotected by a quick pass through reservoir solution supplemented with 20% v/v glycerol before flash cryocooling in a cold (100 K) stream of nitrogen gas. Diffraction data were recorded from a single crystal of SeMet VSV_NJ_ M at a wavelength of 0.9803 Å, to maximize the selenium anomalous signal, on ESRF beamline ID23EH1. Diffraction data were processed using XDS [55] and SCALA [56] as implemented by the xia2 automated data processing package (Winter *et al.*, in preparation).

### Structure solution and refinement

The structures of VSV_NJ_ and LBV M were solved at 1.83 Å and 3.0 Å resolution by single- and multiple-wavelength anomalous dispersion analysis using the diffraction data described above and elsewhere [54], respectively. For both, selenium atoms were located and their positions refined using SHELXD [57] and SHARP [58] as implemented by autoSHARP [59]. For VSV_NJ_ M, electron density maps were solvent-flatted using SOLOMON [60] and DM [61]. The structure was traced by manually placing VSV_Ind_ M^th^ (PDB ID 1LG7; [19]) into electron density and subsequently rebuilding in COOT [62]. For LBV M, the experimental map was solvent flattened and an initial partial model traced using cycles of automatic building in RESOLVE and restrained refinement in REFMAC5 [63]–[65] followed by manual rebuilding in COOT. The initial LBV M model was placed into the high-resolution (2.75 Å) native data by rigid-body refinement in REFMAC5. Final TLS+restrained refinement of both structures was performed in REFMAC5 [66], earlier refinement of LBV M having been performed using BUSTER/TNT [67]. The MolProbity server [68] and the validation tools present in COOT informed the refinement of both structures. Refinement statistics are shown in Table 1 and final refined coordinates and structure factors have been deposited with the PDB with accession IDs 2w2r (VSV_NJ_ M) and 2w2s (LBV M).

### Structure analysis

Superpositions and structure-based alignment of VSV and LBV M were performed using SSM [69] and MUSTANG [70]. Vesiculovirus and lyssavirus M protein sequences were aligned using MUSCLE [71] and sequence alignment figures were produced with the assistance of JalView [72] and Inkscape (http://www.inkscape.org). Molecular graphics were produced using PyMOL (DeLano Scientific LLC).

## Supporting Information

Figure S1 Anomalous scattering allows positive identification of residues bound to the globular domains of VSV_NJ_ and LBV M. Anomalous difference density co-located with the Se atoms in the interacting regions of SeMet-labelled (A) VSV_NJ_ M (carbon atoms pink) and (B) LBV M (carbon atoms violet) allowed unambiguous identification of the interacting sequences. For both, final refined coordinates are shown in experimental electron density calculated after solvent flattening (blue, 1 σ). Anomalous difference maps calculated using anomalous differences from the peak wavelength and using solvent-flattened phases are shown in orange (8 σ).
(5.38 MB PNG)

Figure S2 SDS PAGE analysis of M crystals confirms that the proteins have not been degraded during crystallisation. (A) VSV_NJ_ M, molecular weight 26.2 kDa. (B) LBV M, molecular weight 23.0 kDa. Molecular size markers are shown in kDa. Crystals were prepared for SDS-PAGE analysis by removing all mother-liquor surrounding the crystals using a fine paper wick (Hampton Research), washing the crystals in situ with 0.3 µL reservoir solution, wicking away the reservoir solution, and then dissolving the washed crystals in 0.6 µL 8 M urea. Dissolved crystals were diluted to 5 µL in ultra-pure water, 2 µL of 4× SDS-PAGE loading buffer was added (Invitrogen) and the sample heated to 95°C for 5 min before being loaded onto a 10% w/v polyacrylamide NuPAGE gel that was run in MES buffer according to the manufacturer's instructions (Invitrogen). Protein bands were visualized with SafeStain (Invitrogen).
(0.35 MB PNG)

Figure S3 Analytical gel filtration of full-length and N-terminally truncated LBV M. While the elution of LBV M lacking the N-terminal 45 residues (Δ 1–45 LBV M) is consistent with its mass, full-length LBV M elutes considerably later than expected for a monomer (but earlier than expected for a dimer). This is consistent with the first 45 residues of LBV M adopting an extended/disordered conformation in solution. Δ 1–45 LBV M was cloned from reverse-transcribed LBV genomic DNA [54] into pOPINF (thereby adding an N-terminal His_6_ affinity tag and 3C cleavage site for removal of said tag) by InFusion ligation-independent cloning [74] using the PCR primers 5′-AAGTTCTGTTTCAGGGCCCGGGCAAAGAGAATGTTAGAAACTTTTGTATAAATGG-3′ (forward) and 5′-ATGGTCTAGAAAGCTTTATTCCAACAGAAGTGAAGTGTTCTCATCTTC-3′ (reverse). Δ 1–45 LBV M was expressed in E. coli Rosetta(DE3)pLysS using auto-induction medium as described [75]. Lysis and initial Ni-NTA purification was as described for SUMO-tagged, full-length LBV M [54]. The eluate was diluted to reduce the imidazole concentration to 33 mM using gel filtration buffer (25 mM Hepes pH 8.0, 100 mM NaCl, 5 mM DTT, 0.1 mM ZnCl_2_) and then treated with 200 µg of 3C protease overnight at 4°C [76]. Following cleavage, 2 mL of Ni-NTA Sepharose beads (GE Healthcare) were added to the mixture, incubated for a further hour on ice and applied to a disposable chromatography column (Econopak, Bio-Rad). The flow-through was collected, concentrated and applied to a Superdex 75 column (HiLoad 16/60, GE Healthcare) equilibrated in gel filtration buffer. Peak fractions were pooled, concentrated and the protein identity verified by mass spectroscopy. Full-length and Δ 1–45 LBV M were concentrated to ∼0.3 mg/mL as estimated by A_280_ and theoretical extinction coefficients using 5 kDa molecular weight cut-off micro-concentrators (Vivascience). Samples (100 or 150 µL) were applied to a Superdex S75 10/300 GL gel filtration column (GE Healthcare) pre-equilibrated in 25 mM HEPES pH 8.0, 100 mM NaCl, 5 mM DTT, 0.1 mM ZnCl_2_ and eluted at 0.5 mL/min. The column was calibrated using the molecular size standards conalbumin (75 kDa), ovalbumin (43 kDa), carbonic anhydrase (29 kDa), ribonuclease A (13.7 kDa) and aprotinin (6.5 kDa) from the low molecular weight gel filtration calibration kit (GE Healthcare). K_av_ was calculated using the equation K_av_ = (V_E_−V_0_)/(V_C_−V_0_) where V_E_ is the elution volume in mL, V_0_ is the void volume in mL as measured by the elution of blue dextran, and V_C_ is the geometric volume of the column (24 mL). The elution profiles of full-length and Δ 1–45 LBV M were not significantly perturbed by increasing the concentration of NaCl to 500 mM, nor was the elution profile of full-length LBV M perturbed by the absence of 0.1 mM ZnCl_2_ or by the presence of 0.1 mM EDTA (not shown).
(0.08 MB PNG)

Figure S4 Stereogram of LBV M packing in the crystal. Residues 31–37 (violet), which interact with the globular domain of LBV M (green) may come from one of three molecules, related by the following symmetry operators: [1+x−y, 1−y, 1−z] (red; 22.6 Å from C^α^Glu37 to C^α^Glu48), [1+y, 1−x+y, −1/6+z] (orange; 32.4 Å from C^α^Glu37 to C^α^Glu48), [1−x+y, 1−x, −1/3+y] (blue; 35.5 Å from C^α^Glu37 to C^α^Glu48). The red molecule is the only one that makes additional crystal contacts with the globular domain, this interaction burying 970 Å^2^ of surface area. Distances between the C^α^ atom of residue 37 of the bound polyproline motif and the residue 48 C^α^ atoms of the symmetry-related molecules are shown as dotted lines. For clarity only selected symmetry-related molecules are shown (grey). (A) and (B) represent two orthogonal views.
(4.30 MB PNG)

Figure S5 The interaction between the β2–β3 loop of VSV_NJ_ M and the bound peptide. Residues of the globular domain (carbon atoms green) and the N-terminal interacting residues (carbon atoms pink) are shown as sticks, and the molecular surface of the globular domain is shown in white, highlighting that mutation of AVLA (121–124) with DKQQ would not disrupt hydrophobic pocket that binds F46. The side chains of A121 and A124 point away from the bound peptide. While the side chain of L123 forms part of the shallow hydrophobic cleft into which M51 binds, the L123Q mutation would not prohibit peptide binding as there is ample room for the surface-exposed side chain to move away; indeed it is possible that the hydrophobic face of the glutamine side chain amide might replace the hydrophobic leucine side chain and form part of the binding pocket. The substitution of V122, which interacts with M48 of the bound peptide, would equally not preclude binding. In VSV_Ind_, the strain for which the MDKQQ mutant was generated, M48 is replaced with the shorter hydrophobic amino acid valine. The presence of this shorter side chain on the peptide would allow sufficient space for V122 to be replaced by lysine without severely disrupting binding.
(0.77 MB PNG)
