# Supplementary figures and images for: Rhabdovirus Matrix Protein Structures Reveal a Novel Mode of Self-Association
Source: PLoS Pathog. 2008 Dec 26;4(12):e1000251. doi: 10.1371/journal.ppat.1000251 (PMC2603668; doi:10.1371/journal.ppat.1000251)

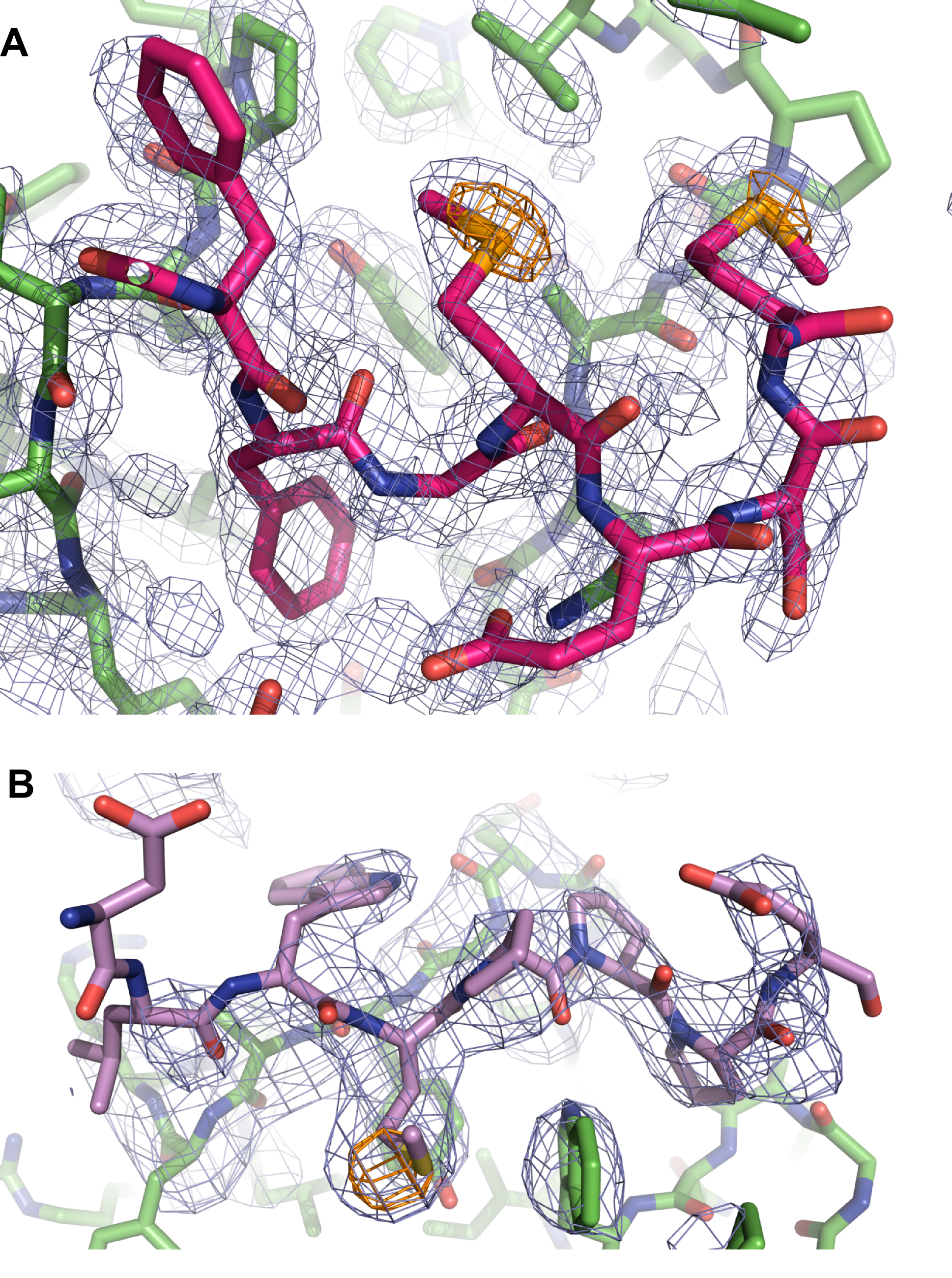

Supplement: Figure S1 — Anomalous scattering allows positive identification of residues bound to the globular domains of VSVNJ and LBV M. Anomalous difference density co-located with the Se atoms in the interacting regions of SeMet-labelled (A) VSVNJ M (carbon atoms pink) and (B) LBV M (carbon atoms violet) allowed unambiguous identification of the interacting sequences. For both, final refined coordinates are shown in experimental electron density calculated after solvent flattening (blue, 1 σ). Anomalous difference maps calculated using anomalous differences from the peak wavelength and using solvent-flattened phases are shown in orange (8 σ). (5.38 MB PNG) [file ppat.1000251.s001.png]

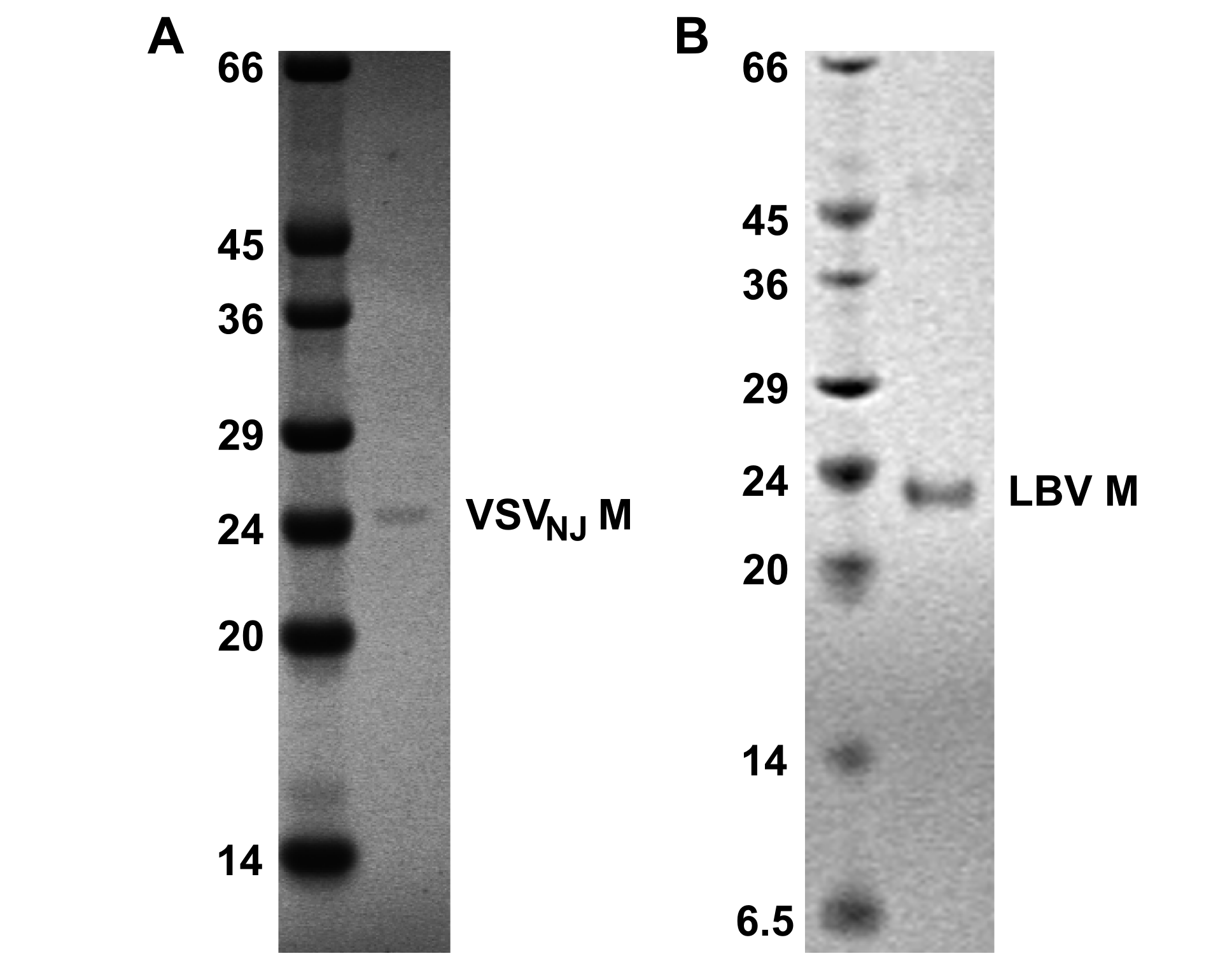

Supplement: Figure S2 — SDS PAGE analysis of M crystals confirms that the proteins have not been degraded during crystallisation. (A) VSVNJ M, molecular weight 26.2 kDa. (B) LBV M, molecular weight 23.0 kDa. Molecular size markers are shown in kDa. Crystals were prepared for SDS-PAGE analysis by removing all mother-liquor surrounding the crystals using a fine paper wick (Hampton Research), washing the crystals in situ with 0.3 µL reservoir solution, wicking away the reservoir solution, and then dissolving the washed crystals in 0.6 µL 8 M urea. Dissolved crystals were diluted to 5 µL in ultra-pure water, 2 µL of 4× SDS-PAGE loading buffer was added (Invitrogen) and the sample heated to 95°C for 5 min before being loaded onto a 10% w/v polyacrylamide NuPAGE gel that was run in MES buffer according to the manufacturer's instructions (Invitrogen). Protein bands were visualized with SafeStain (Invitrogen). (0.35 MB PNG) [file ppat.1000251.s002.png]

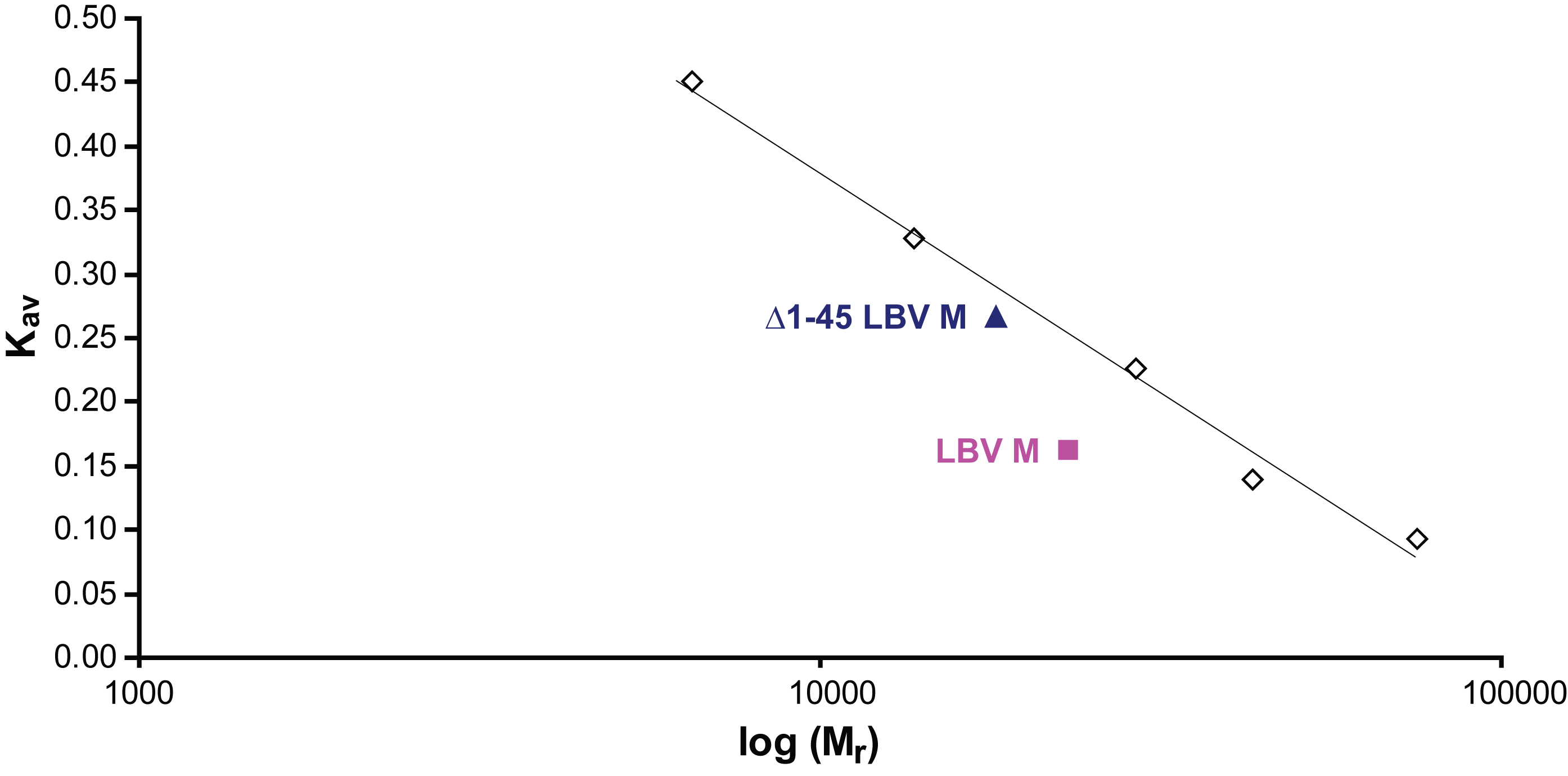

Supplement: Figure S3 — Analytical gel filtration of full-length and N-terminally truncated LBV M. While the elution of LBV M lacking the N-terminal 45 residues (Δ 1–45 LBV M) is consistent with its mass, full-length LBV M elutes considerably later than expected for a monomer (but earlier than expected for a dimer). This is consistent with the first 45 residues of LBV M adopting an extended/disordered conformation in solution. Δ 1–45 LBV M was cloned from reverse-transcribed LBV genomic DNA [54] into pOPINF (thereby adding an N-terminal His6 affinity tag and 3C cleavage site for removal of said tag) by InFusion ligation-independent cloning [74] using the PCR primers 5′-AAGTTCTGTTTCAGGGCCCGGGCAAAGAGAATGTTAGAAACTTTTGTATAAATGG-3′ (forward) and 5′-ATGGTCTAGAAAGCTTTATTCCAACAGAAGTGAAGTGTTCTCATCTTC-3′ (reverse). Δ 1–45 LBV M was expressed in E. coli Rosetta(DE3)pLysS using auto-induction medium as described [75]. Lysis and initial Ni-NTA purification was as described for SUMO-tagged, full-length LBV M [54]. The eluate was diluted to reduce the imidazole concentration to 33 mM using gel filtration buffer (25 mM Hepes pH 8.0, 100 mM NaCl, 5 mM DTT, 0.1 mM ZnCl2) and then treated with 200 µg of 3C protease overnight at 4°C [76]. Following cleavage, 2 mL of Ni-NTA Sepharose beads (GE Healthcare) were added to the mixture, incubated for a further hour on ice and applied to a disposable chromatography column (Econopak, Bio-Rad). The flow-through was collected, concentrated and applied to a Superdex 75 column (HiLoad 16/60, GE Healthcare) equilibrated in gel filtration buffer. Peak fractions were pooled, concentrated and the protein identity verified by mass spectroscopy. Full-length and Δ 1–45 LBV M were concentrated to ∼0.3 mg/mL as estimated by A280 and theoretical extinction coefficients using 5 kDa molecular weight cut-off micro-concentrators (Vivascience). Samples (100 or 150 µL) were applied to a Superdex S75 10/300 GL gel filtration column (GE Healthcare) pre-equilibrated in 25 mM HEPES pH 8.0, [file ppat.1000251.s003.png]

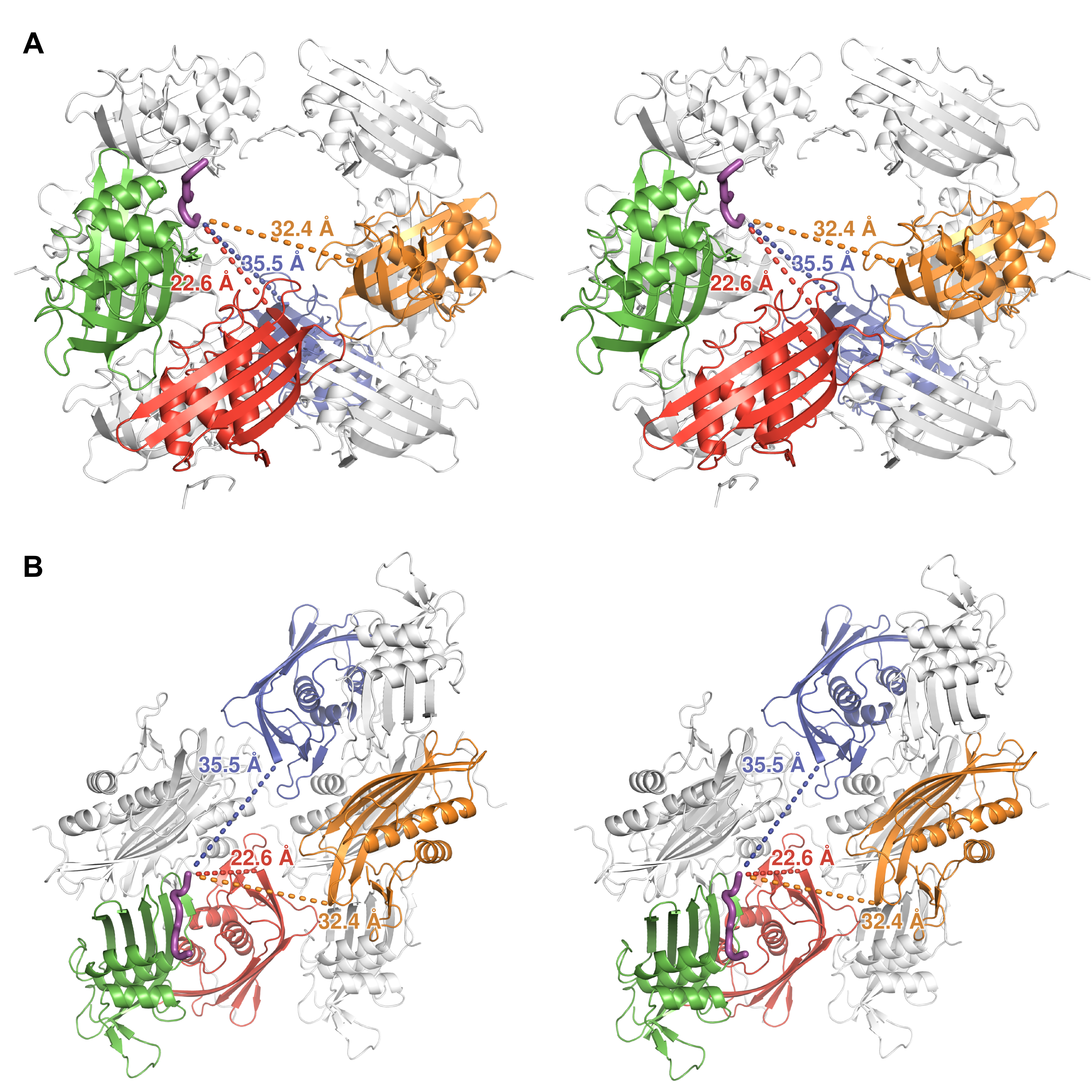

Supplement: Figure S4 — Stereogram of LBV M packing in the crystal. Residues 31–37 (violet), which interact with the globular domain of LBV M (green) may come from one of three molecules, related by the following symmetry operators: [1+x−y, 1−y, 1−z] (red; 22.6 Å from CαGlu37 to CαGlu48), [1+y, 1−x+y, −1/6+z] (orange; 32.4 Å from CαGlu37 to CαGlu48), [1−x+y, 1−x, −1/3+y] (blue; 35.5 Å from CαGlu37 to CαGlu48). The red molecule is the only one that makes additional crystal contacts with the globular domain, this interaction burying 970 Å2 of surface area. Distances between the Cα atom of residue 37 of the bound polyproline motif and the residue 48 Cα atoms of the symmetry-related molecules are shown as dotted lines. For clarity only selected symmetry-related molecules are shown (grey). (A) and (B) represent two orthogonal views. (4.30 MB PNG) [file ppat.1000251.s004.png]

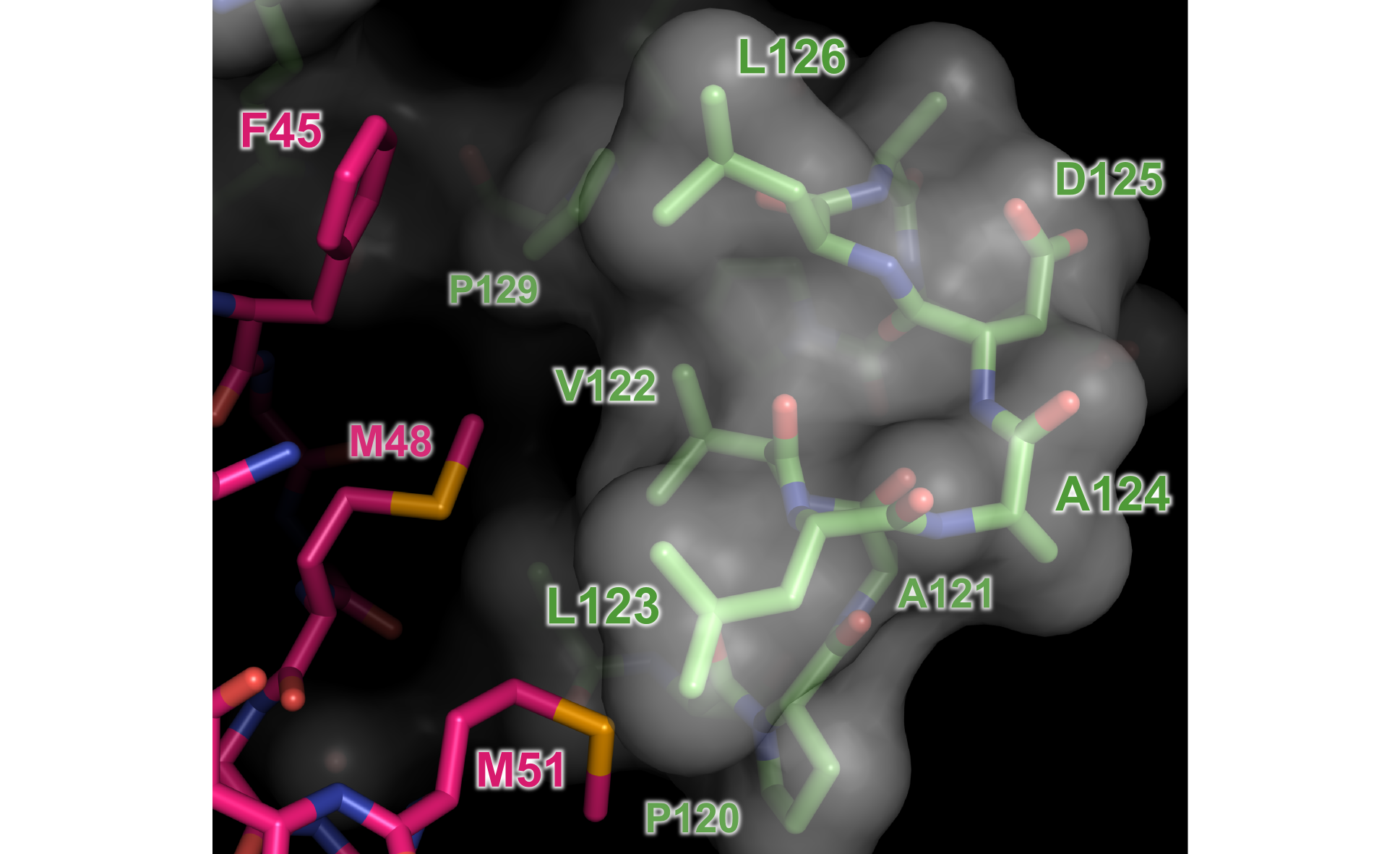

Supplement: Figure S5 — The interaction between the β2–β3 loop of VSVNJ M and the bound peptide. Residues of the globular domain (carbon atoms green) and the N-terminal interacting residues (carbon atoms pink) are shown as sticks, and the molecular surface of the globular domain is shown in white, highlighting that mutation of AVLA (121–124) with DKQQ would not disrupt hydrophobic pocket that binds F46. The side chains of A121 and A124 point away from the bound peptide. While the side chain of L123 forms part of the shallow hydrophobic cleft into which M51 binds, the L123Q mutation would not prohibit peptide binding as there is ample room for the surface-exposed side chain to move away; indeed it is possible that the hydrophobic face of the glutamine side chain amide might replace the hydrophobic leucine side chain and form part of the binding pocket. The substitution of V122, which interacts with M48 of the bound peptide, would equally not preclude binding. In VSVInd, the strain for which the MDKQQ mutant was generated, M48 is replaced with the shorter hydrophobic amino acid valine. The presence of this shorter side chain on the peptide would allow sufficient space for V122 to be replaced by lysine without severely disrupting binding. (0.77 MB PNG) [file ppat.1000251.s005.png]
